# Crop immunity against viruses: outcomes and future challenges

**DOI:** 10.3389/fpls.2014.00660

**Published:** 2014-11-21

**Authors:** Valérie Nicaise

**Affiliations:** Fruit Biology and Pathology, Virology Laboratory, Institut National de la Recherche Agronomique, University of BordeauxUMR 1332, Villenave d’Ornon, France

**Keywords:** plant virus, *R* gene, recessive resistance, gene silencing, systemic acquired resistance, PAMP-triggered immunity, crop improvement

## Abstract

Viruses cause epidemics on all major cultures of agronomic importance, representing a serious threat to global food security. As strict intracellular pathogens, they cannot be controlled chemically and prophylactic measures consist mainly in the destruction of infected plants and excessive pesticide applications to limit the population of vector organisms. A powerful alternative frequently employed in agriculture relies on the use of crop genetic resistances, approach that depends on mechanisms governing plant–virus interactions. Hence, knowledge related to the molecular bases of viral infections and crop resistances is key to face viral attacks in fields. Over the past 80 years, great advances have been made on our understanding of plant immunity against viruses. Although most of the known natural resistance genes have long been dominant R genes (encoding NBS-LRR proteins), a vast number of crop recessive resistance genes were cloned in the last decade, emphasizing another evolutive strategy to block viruses. In addition, the discovery of RNA interference pathways highlighted a very efficient antiviral system targeting the infectious agent at the nucleic acid level. Insidiously, plant viruses evolve and often acquire the ability to overcome the resistances employed by breeders. The development of efficient and durable resistances able to withstand the extreme genetic plasticity of viruses therefore represents a major challenge for the coming years. This review aims at describing some of the most devastating diseases caused by viruses on crops and summarizes current knowledge about plant–virus interactions, focusing on resistance mechanisms that prevent or limit viral infection in plants. In addition, I will discuss the current outcomes of the actions employed to control viral diseases in fields and the future investigations that need to be undertaken to develop sustainable broad-spectrum crop resistances against viruses.

## INTRODUCTION

As obligatory intracellular parasites, plant viruses depend on the host machinery to multiply and invade their hosts. In their simplest form, viruses consist of a DNA or RNA genomic segment encoding only few genes and encapsided into a protein shell, called the capsid. Because of natural physical barriers (cuticle, cell wall), viruses are delivered into plant cells through wounds or through the action of vectors (insects, nematodes, fungi) that feed on or infect the plants. Following entry into a host cell and genome decapsidation, the infectious cycle includes translation and replication of the viral genome, assembly of progeny virus particles, generalized invasion of the host through cell-to-cell and long-distance movements of viral particles or ribonucleoprotein complexes and finally, transmission to new hosts by vectors. In some cases, transmission to the following generation of host plant is also observed as a result of seed infection. In 2012, the International Committee on Taxonomy of Viruses reported 92 genera of plant viruses of which 82 were assigned in 21 different families (King et al., 2012).

Plant infection by viruses causes physiological disorders responsible for plant diseases of economic and agronomic significance in many crops. Widely employed in fields, the use of plant varieties carrying genetic resistances constitutes the most effective, economical and ecological measure to control viral infections. The last decades have seen substantial advances on the molecular dialog between viral pathogens and their plant hosts, bringing new strategies directly exploitable in crop improvement programs. Nevertheless, the spread of crop viral pests has increased dramatically in recent years. Globalization, trade and climate change, as well as reduced resilience in production systems due to decades of agricultural intensification have all played a part.

This review intends: (i) to provide a brief overview of severe virus-associated plant diseases and their impact on crop production, (ii) to summarize prophylactic strategies employed to control viral epidemics in fields, (iii) to bring an update on current knowledge about plant resistances against viruses, (iv) to present the technical approaches currently employed in crop improvement programs, and (v) to discuss how antiviral mechanisms based on PAMP-triggered immunity (PTI) could be the source of novel plant resistances in fields.

## WHAT IS THE REAL IMPACT OF VIRAL DISEASES ON CROPS?

Management of plant virus diseases is a matter of vital importance and concern to farmers, horticulturists, foresters, manufacturers, as well as consumers. It is well-established that virus diseases in different crops cause enormous losses all over the world in terms of quantity and/or quality of products. Although it’s very difficult to put a clear figure on the financial impact of plant viruses in agriculture, the yield losses that can be ascribed to plant viruses are estimated to cost worldwide more than $30 billion annually (Sastry and Zitter, 2014).

Amongst the most damaging virus-associated threats, strains of *Cassava mosaic begomoviruses* cause more than 25 million tons of losses per year in Africa, India, and Sri Lanka (Legg and Thresh, 2000; Calvert and Tresh, 2002; Thresh and Cooter, 2005). Because the Cassava crop represents the daily staple for more than 500 million people all over the world, epidemics are often associated to famine events (Legg, 1999; Legg and Thresh, 2000; Calvert and Tresh, 2002). *Potato leafroll polerovirus* is responsible for an annual potato loss of $100 millions in the US and #x000A3;30–50 millions in UK (Wale et al., 2008; Sastry and Zitter, 2014). The losses in citrus tree cultures attributed to *Citrus Tristeza closterovirus* (CTV) were estimated to over 100 million trees worldwide (Moreno et al., 2008; Harper, 2013). Barley yellow dwarf is the most widely distributed viral disease of cereals, affecting oats, rice, barley, maize, and wheat. It is caused by *Barley yellow dwarf luteovirus*, that costs producers in UK alone about £10 million a year in lost production (Ordon et al., 2009; Sastry and Zitter, 2014). It is estimated that the international costs of managing the Sharka disease [caused by *Plum pox potyvirus* (PPV)] since the 1970s have exceeded 10 billion euros (Cambra et al., 2006). Viruses affecting rice cultures result in yield losses estimated at more than $1.5 billion in South-East Asia alone (Abo and Sy, 1998; Hull, 2013; Sasaya et al., 2013). Discovered in Ghana in the middle of the 20th century, *Cacao swollen shoot badnavirus* is currently endemic in Togo, Ghana, and Nigeria. Over 200 million trees have already been eradicated, representing the most costly effort of any country in the world against a viral plant disease (Dzahini-Obiatey et al., 2010).

It’s important to mention that diseases of perennial and fruit crops not only lead to the loss of the crop but the loss of time and cost in bringing the trees to bearing, the losses of other crops that could have been grown on the land during that time, and the differences in the value of the land with and without a productive orchard. Moreover, virus-associated losses in fields are highly under-estimated as some viral infections are asymptomatic alone but contribute in a synergic manner to damages due to attacks from other pathogens (Hull, 2013). More importantly, viruses have been described causing half of the reported emerging infectious diseases from plants (Anderson et al., 2004). Hence, viruses represent serious crop threats, responsible for considerable agronomical losses, global food problem, and human life overall all over the world.

## PROPHYLACTIC MEASURES IN FIELDS

**FIGURE 1:**
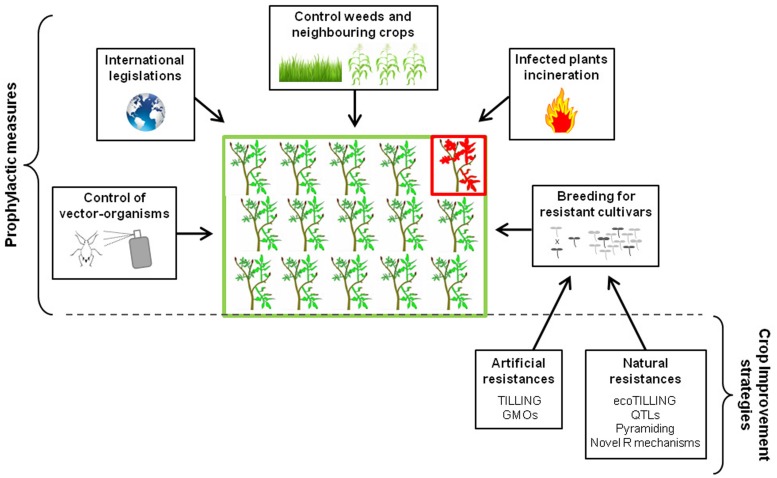
**Prophylactic measures and main crop improvement strategies employed to control plant viral diseases**.

Because viral agents are obligate intracellular parasites, curative treatments of virus infections are impossible, making viral diseases very difficult to control in fields. Prophylactic control measures are therefore crucial in combating epidemics on crops. They consist mainly on combining cultural practices, biosecurity measures and organism-vector management (**Figure 1**).

### PERFORM REGULAR INSPECTION FOR THE PRESENCE OF VIRAL PATHOGENS

In this domain, the rising up of molecular biology techniques combined to continuous characterization of new etiological agents improved significantly the sensitivity, the specificity and the rapidity required to an accurate diagnosis of plant pathogenic viruses (Boonham et al., 2014). The reliability of the available diagnosis tests is a key point in viral disease management in fields, as infected plants need to be eradicated as fast as possible to minimize the virus spread.

### MONITOR ORGANISM-VECTOR POPULATIONS

Plant viruses need to be transmitted by an organism-vector (insects, nematodes, zoosporic endoparasites) for their plant-to-plant spread. Hence, viral diseases can be efficiently controlled by limiting the populations of their vectors with the applications of appropriate pesticides. The use of non-host “trap plants” may be also considered to attract vectors to reduce the number of individuals feeding on the crop of interest and thus, the transmission of the disease (Bragard et al., 2013).

### SET UP A RIGOROUS CONTROL PROGRAM ON WEEDS AND OTHER HOST PLANTS IN THE VICINITY OF THE FIELD

Epidemics often arise from new viruses or new variants of classic viruses that spilled over from reservoir species to crops. Although this phenomenon results from a complex evolutionary process in which the main players are ecological factors, virus genetic plasticity and host factors, viral diseases can be controlled by managing the spatial structure and composition of field parcels, which impacts resistance durability (Elena et al., 2011; Fabre et al., 2012).

### RESPECT THE PHYTOSANITARY MEASURES DECREED BY VARIOUS INTERNATIONAL COMMISSIONS

Minimizing viral epidemics involves the respect of international legislations concerning worldwide trade of virus-free plant material, which applies to any development stage of a plant that can be carrier of viruses (seeds or fruit stones, grafts, rootstocks, seedlings, flowers,...), as well as manipulation of decontaminated horticultural tools.

### USE CROP CULTIVARS THAT ARE RESISTANT TO VIRUSES

The use of genetically resistant plants is one of the most efficient, sustainable and frequently employed strategies to control virus infections in fields. For centuries, it has involved plants selected by breeders for their agronomic proprieties combined to the absence of disease symptoms. However, from the middle of the 20th century, plant improvement programs capitalize strongly on the knowledge associated to plant–virus interactions to develop resistant varieties exploitable in agriculture.

## WHAT DO WE KNOW ABOUT PLANT IMMUNITY AGAINST VIRUSES?

**FIGURE 2:**
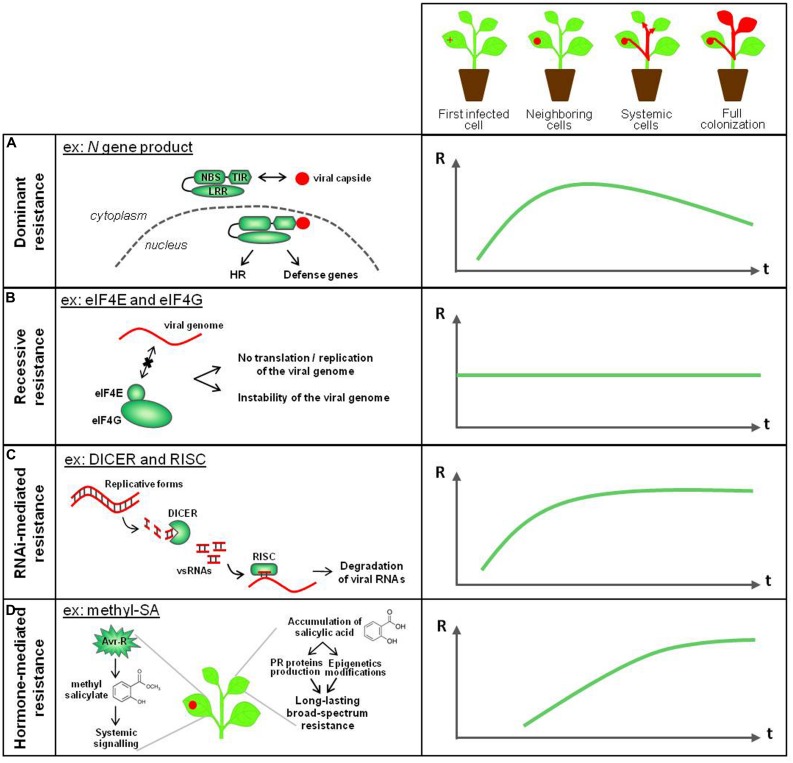
**Known antiviral immune mechanisms in plants.** Plant resistance mechanisms against viruses are complementary in terms of plant defense timing, location (from the first infected cell to the generalized colonization) and targeting the virus-derived molecules (genome or proteins from viruses). **(A)** NBS-LRR dominant resistance relies on the interaction between an avirulence factor and a specific *R* gene product, and is effective several days after the virus entry into the plant. The HR-associated phenomenon confines the viral pathogen in the infected and neighboring cells. **(B)** Recessive resistance, that corresponds to the absence of appropriate host factors that are required for the virus cycle, is a non-inducible resistance, passive and effective throughout plant colonization. It confers resistance at the infection step that requires the cellular factor of interest.**(C)** RNA interference (RNAi) targets viral nucleic acids. Once set up after few days, the effectiveness of this defense mechanism increases and spreads to the whole plant through a relay-amplification process. **(D)** Hormone-mediated resistance against viral pathogens is represented here by the role of salicylic acid (SA) and methyl-salicylate (Me-SA) in systemic acquired resistance (SAR). On graphs, R, resistance level; t, infection timing.

Faced with viral attacks, plants defend themselves through several resistance layers, that are complementary in terms of defense timing (at early or late infection steps), location (in the first infected leaf or in systemic tissues) and targeting the virus-derived molecules (the viral genome or the viral proteins; **Figure 2**).

### DOMINANT RESISTANCES

The majority of dominant resistance genes (*R* genes) identified in plant–virus interactions belong to the nucleotide binding site-leucine-rich repeat (NBS-LRR) class, that specifically recognize the viral avirulence (*avr*) gene products, through the establishment of the so-called “gene-for-gene” interaction. Although a direct physical interaction between the *avr* and *R* gene products had originally been suggested, the current understanding favors the more sophisticated “guard hypothesis” model (Soosaar et al., 2005). Many NBS-LRR proteins conferring resistance against viruses have been identified so far, and are classified on the basis of their N-terminal structure, that carries either a Toll–interleukin-1 receptor (TIR) domain or a coiled-coil (CC) domain (Moffett, 2009; De Ronde et al., 2014).

One of the best characterized example is the potato *Rx1* gene that encodes a typical CC-NBS-LRR protein and mediates resistance to *Potato potexvirus X* (PVX) through the recognition of the PVX capsid (CP). Rx1 CC domain has been shown to form a heterodimer with the cellular ranGTPase-activating protein 2 (ranGAP2), interaction required for Rx1 function (Rairdan et al., 2008; Tameling et al., 2010). Although direct interaction between ranGAP2 and PVX CP has not been detected, it has been proposed that ranGAP2 might nonetheless interact with the CP, causing a conformational change perceived by the rest of the protein and leading Rx to switch to an active state (Hao et al., 2013). In another well-characterized *R* gene example, the tobacco *N* gene product, that displays a TIR-NBS-LRR structure, interacts directly with the helicase domain of the replicase of *Tobacco mosaic tobamovirus* (TMV), in an ATP dependent manner (**Figure 2A**; Ueda et al., 2006). Full resistance to TMV requires the N receptor-interacting protein 1 (NRIP1), which is recruited from the chloroplast to the cytoplasm and nucleus and interacts with both viral replicase and N factor (Caplan et al., 2008). In both *Rx1* and *N* cases, the *R* gene product is activated in the cytoplasmic compartment, while its nucleocytoplasmic distribution is required for full functionality (Slootweg et al., 2010; Tameling et al., 2010). The study of *N*- and *Rx*-mediated resistances have led to characterize the R signaling cascade in plant–virus interactions, that include rapid activation of MAP kinases and the action of a molecular chaperone complex composed of SGT1 (*Suppressor of G2 allele of SKP1*), HSP90 (*Heat shock protein*), and RAR1 (*Required for Mla resistance 1*; Azevedo et al., 2006; Botër et al., 2007; Kobayashi et al., 2010; Hoser et al., 2013), whose role seems to both stabilize R factors and mediate their degradation, thereby maintaining a tight cellular balance between defense signaling and attenuation (Kadota and Shirasu, 2012).

Immune events downstream of R protein activation are frequently associated with calcium ion influx, MAPK-mediated signaling, reactive oxygen species (ROS) production, salicylic acid (SA) accumulation, and extensive transcriptional reprogramming. In addition, the activation of *R* genes is most of the time associated with a hypersensitive response (HR), a phenomenon involving the programmed death of the infected and neighboring cells, with the consequences to confine the pathogen in the hypersensitive lesion and to prevent any further pathogen spread in the plant. A functional module that mediates HR against viruses (as well as non-viral pathogens) requires the interaction of two lipases, EDS1 (*Enhanced disease susceptibility 1*) and PAD4 (*Phytoalexin deficient 4*) with the protein SAG101 (*Senescence-associated gene 101*; Liu et al., 2002; Marathe et al., 2002; Wagner et al., 2013). In *Arabidopsis*, the EDS1/PAD4/SAG101 complex regulates *HRT*-mediated resistance against *Turnip crinkle carmovirus* (Zhu et al., 2011). Recent insights into R protein downstream signaling indicate that the resistance process and the HR-programmed cell death are distinct physiological pathways, although both can work in concert (Bendahmane et al., 1999; Bai et al., 2012). Meanwhile *R* gene mediated defense is taking place locally, it also induces defense signaling in distally located tissues, called systemic acquired resistance (SAR), a mechanism that has been demonstrated for both *N* and *Rx1* genes (Delaney et al., 1994; Liu et al., 2010) and that is described in the Section “Plant Hormone-Mediated Resistance” of this review.

In contrast with the structure of the classical *R* genes, the *RTM* genes (for *Restricted TEV Movement*) were the first cloned non-NBS-LRR dominant resistance genes against viruses. Genetic characterization of natural *Arabidopsis* accessions and mutants showed that at least five dominant *RTM* genes are involved in resistance to *Tobacco etch potyvirus* (TEV), *Lettuce mosaic potyvirus* (LMV), and PPV (Cosson et al., 2012). It has been recently proposed that RTM members may form a phloem-resident multiprotein complex involved in the resistance mechanisms to block the long-distance movement of potyviruses (Cosson et al., 2010a,b). No induction of HR or production of SA has been detected, in contrast with NBS-LRR mediated resistance responses (Mahajan et al., 1998). Another example of a non-NLS-LRR dominant resistance conferring resistance to TMV comes from the tomato *Tm-1* gene, which encodes a protein with a TIM-barrel like structure, that interacts directly with the viral replicase, strongly impairing the viral genome replication (Ishibashi and Ishikawa, 2013).

### RESISTANCE RECESSIVES

Around one-half of the approximately 200 known resistance genes that target plant viruses are recessively inherited (Diaz-Pendon et al., 2004), suggesting that this form of resistance is more common for viruses than for other plant pathogens. The use of such genes is therefore a very important tool in breeding programs to control plant diseases caused by pathogenic viruses.

Recessive resistances are often achieved through the absence of appropriate host factors that are required for the virus to complete its biological cycle. Over the last decade, a large number of recessive resistance genes have been cloned from crop species and shown to encode eukaryotic initiation factors (eIFs) belonging to the eIF4E and eIF4G families (**Figure 2B**; Truniger and Aranda, 2009; Wang and Krishnaswamy, 2012; Julio et al., 2014; Revers and Nicaise, 2014). These factors belong to the cellular 43S complex, that recruit both mRNAs and ribosomes before the process of translation (Pestova et al., 2001) and are hypothesized to act as susceptibility factors recruited during the replication/translation steps of the viral genome. Firstly highlighted for successful potyvirus infection, the role of eIF4E and eIF4G have been quickly extended to other plant virus families, involving bymoviruses, cucumoviruses, ipomoviruses, sobemoviruses, carmoviruses, and waikiviruses (Revers and Nicaise, 2014), suggesting that they contribute to a broad mechanism of plant susceptibility to viruses. An intriguing aspect of eIF4E- and eIF4G-mediated resistances is that they cover a diverse range of resistance phenotypes. Although in many investigated cases they govern complete qualitative resistance, they have also been shown in some instances to provide partial resistance or to be components of partial or polygenic resistances (Caranta et al., 1997; Nicaise et al., 2003; Acosta-Leal and Xiong, 2008; Charron et al., 2008). Interestingly, ectopic overexpression of an *eIF4E* resistance gene generates dominant potyvirus resistance in tomato and potato crops (Kang et al., 2007; Cavatorta et al., 2011), probably through the fact that the abundance of the “resistant eIF4E” makes the endogenous “susceptible eIF4E” inaccessible for its recruitment by viruses. Other components belonging to the translational machinery were demonstrated to be required for virus multiplication in *Arabidopsis thaliana* and *Nicotiana benthamiana*, including the translation initiation factor 4B (eIF4B), the translation elongation factors 1A and 1B (eEF1A and eEF1B) and the poly(A)-binding proteins (PABPs) 2, 4, and 8 (Sasvari et al., 2011; Patarroyo et al., 2012; Hwang et al., 2013; Li et al., 2014b). These results suggest that crop genes encoding these translation factors may lead to new resistance sources that need to be explored for viral disease control.

Other susceptible recessive resistance genes which are not encoding translation initiation factors have been identified through the analysis of mutants collections and/or natural cultivated or wild species. A positional cloning strategy exploring barley natural variability revealed recently the key role of the PDI5-1 (*Protein disulfide isomerase like 5-1*) protein in the recessive resistance to bymoviruses (Yang et al., 2014). Another recessive resistance gene named *ra* blocking vascular transport of *Potato potyvirus A* (PVA) was genetically characterized in potato (Hämäläinen et al., 2000). In addition, from the perspective of identifying new resistance sources, exploring *Arabidopsis* genetic diversity has provided original insights into the genes involved in plant–virus interactions, which could be used as potentially resistance sources against viruses. For example, the recessive resistance genes *rlm1* and *rpv1*, conferring resistance to LMV and PPV respectively, map in a genomic region containing no translation factor genes (Revers et al., 2003; Decroocq et al., 2006). The lack of co-segregation with *eIF4E* or *eIF4G* genes is also true for *dstm1* mediating TMV resistance (Serrano et al., 2008) and *sha3*, a major quantitative trait locus (QTL) contributing to systemic resistance against PPV (Pagny et al., 2012). Very recently, data revealed that the gene *rwm1* conferring resistance to *Watermelon mosaic potyvirus* in *Arabidopsis* acts at an early stage of infection by impairing viral accumulation in initially infected leaf tissues and encodes a conserved nucleus-encoded chloroplast phosphoglycerate kinase (Ouibrahim et al., 2014). In the case of *tom1* and *tom2A Arabidopsis* mutants, TMV accumulation is suppressed in single cells. After further characterization, it appears that both genes encode transmembrane proteins localized in the tonoplast and are required for tobamovirus replication (Ishibashi et al., 2012). Consistent with the fact that viral replication complexes associate with host intracellular membranes (Hull, 2013), prospective studies identified many membrane-associated components as key factors required for plant infection success, providing new candidates for novel genetic sources of crop resistances (Diaz et al., 2010; Hyodo et al., 2013; Barajas et al., 2014a,b).

### RNA INTERFERENCE MEDIATED-RESISTANCE

Over the past decades, RNA interference (RNAi; also called gene silencing) has been recognized as an evolutionarily conserved process in most eukaryotes, that is triggered by double-stranded RNAs (dsRNAs). These dsRNAs are processed by ribonuclease III-type DICER-like (DCL) enzymes into small RNAs (sRNAs), 21–24 nucleotides in length, that are incorporated into an RNA-induced cytoplasmic silencing complex (RISC), whose key catalytic component corresponds to one member of the ARGONAUTE (AGO) protein family. Once integrated into the RISC, sRNAs base-pair to their target-mRNA and induce their cleavage (Bologna and Voinnet, 2014). In plants, silencing pathways are particularly diverse and partially overlapping. At least, three basic processes can be distinguished: cytoplasmic RNA silencing (or post-transcriptional gene silencing; PTGS) mediated by small interfering RNAs (siRNAs), silencing-mediated by plant-encoded microRNAs (miRNAs) and transcriptional gene silencing (TGS) mediated by siRNA-directed methylation of DNA and histone proteins. Key components of these RNA silencing pathways have been shown to have an important protective role against invading viral pathogens (Bologna and Voinnet, 2014).

Most plant viruses have RNA genome that commonly contain double-stranded secondary structure elements and/or produce dsRNA intermediates via the action of viral RNA-dependent RNA polymerases (RDRs) during the replication step. These molecules are targeted by the RNA-silencing machinery to produce virus-derived small RNAs (vsRNAs; Pumplin and Voinnet, 2013; **Figure 2C**). What initially appeared counter-intuitive, infection from DNA viruses produce also dsRNAs, most likely via bidirectional convergent transcription (Aregger et al., 2012). The integration of vsRNAs in the RISC leads to the sequence-specific degradation of viral nucleic acids, as well as the generation of a mobile-silencing signal, which spreads between cells through plasmodesmata and over long-distances via the phloem, through a relay-amplification process involving host RDRs (Pumplin and Voinnet, 2013). This process activates RNA silencing in non-infected cells and is notably responsible for the plant recovery phenomenon. Given gene silencing induces immune mechanisms highly specific to the pathogen, it is commonly accepted that RNAi is classified into plant adaptive immunity (Voinnet, 2001; Waterhouse et al., 2001).

Encoded by multigene families, plant DCLs, RDRs and AGOs are often specialized in the production and function of the distinct sRNA classes. Despite its universality, current knowledge remains incompletely understood, as it relies mostly on reverse genetic studies conducted in *Arabidopsis*. This notwithstanding, it appears clear that specific RNAi-associated components are involved in silencing antiviral functions. Thus, DCL4 is the major enzyme for generating RNA virus-derived vsRNAs, even if DCL2 can substitute for DCL4 to some extent (Blevins et al., 2006; Deleris et al., 2006; Qu et al., 2008; Jakubiec et al., 2012). In contrast, all four DCLs (DCL1-4) produce DNA virus-derived vsRNAs (Akbergenov et al., 2006; Moissiard and Voinnet, 2006; Blevins et al., 2011). So far, only AGO1, AGO2, and AGO7 seem to contribute to antiviral RNAi (Morel et al., 2002; Qu et al., 2008; Shivaprasad et al., 2008; Azevedo et al., 2010; Harvey et al., 2011; Jaubert et al., 2011; Wang et al., 2011; Zhang et al., 2012). In particular, RNA viruses-derived vsRNAs seem to be strongly recruited by AGO1 and AGO2 proteins, an observation consistent with the hyper-susceptibility of *ago1 ago2* double mutants (Wang et al., 2011). Antiviral RNAi has also been shown to be dependent on one or more of RDR1, RDR2, and RDR6 for signal initiation and/or amplification (Diaz-Pendon et al., 2007; Qu et al., 2008; Qi et al., 2009; Vaistij and Jones, 2009; Garcia-Ruiz et al., 2010; Wang et al., 2011; Jiang et al., 2012). An increase of susceptibility against RNA viruses is also observed in *Arabidopsis* plants defected for the gene *HEN1* (for HUA ENHANCER 1) encoding a methyltransferase that protects siRNA and miRNA duplexes from degradation (Vogler et al., 2007; Zhang et al., 2012). Interestingly, the natural resistance genes *Ty-1* and *Ty-3* conferring resistance to *Tomato yellow leaf curl begomovirus* (TYLCV) has been recently shown to encode a tomato RDR. Although resistant plants do not show symptoms upon a challenge with TYLCV, low levels of virus are still detectable, a phenomenon characteristic of a virus tolerance more than a real resistance (Verlaan et al., 2013).

Viruses have evolved diverse mechanisms to avoid silencing-mediated resistance, most notably through silencing suppressor activities. Identified for almost all types of plant viruses, silencing suppressors target RNAi pathways at different points and through diverse mechanisms, including the impair of siRNA biogenesis, the defect of siRNA incorporation into the RISC, the degradation of AGOs, the trapping of sRNAs and the suppression of RNAi amplification (reviewed in Burgyán and Havelda, 2011; Pumplin and Voinnet, 2013; Bologna and Voinnet, 2014; Li et al., 2014a). In turn, increasing evidence suggest that plants have evolved by establishing specific defenses against RNA-silencing suppression by pathogens, providing yet another illustration of the never-ending molecular arms race between plant pathogens and their hosts (Pumplin and Voinnet, 2013; Sansregret et al., 2013).

### PLANT HORMONE-MEDIATED RESISTANCE

Plant hormones play important roles in regulating signaling networks involved in plant defenses. Upon pathogen attack, the quantity, composition and timing of the plant hormonal blend produced by the plant depends greatly on the lifestyle and infection strategy of the invading attacker. In the last decades, significant progress has been made in identifying the key components and understanding the role of phytohormones in plant responses to biotic stresses (Robert-Seilaniantz et al., 2011).

During the R-mediated resistance activation (see the Section “Dominant Resistances” in this review), cellular responses elicited at the infection site are emitted to distant non-infected tissues, resulting in a resistance or in reduced susceptibility state that can remain efficient during several weeks (Fu and Dong, 2013). This phenomenon is referred as the SAR (**Figure 2D**). In the case of TMV-triggered HR, the response persists up to 3 weeks during the time plants are protected against not only TMV but also other pathogens (Ross, 1961). How SAR can be sustained for so long is not clear but epigenetic modifications, such as DNA methylation and chromatin remodeling, seem critical to maintain a SAR signal (Spoel and Dong, 2012). Moreover, during a viral infection (in a manner similar to non-viral infections), this long lasting and broad-spectrum disease resistance requires endogenous accumulation of SA, resulting in transcriptional reprogramming of a battery of genes encoding pathogenesis-related (PR) proteins (Tsuda et al., 2008; Yi et al., 2014). The signal emitted by the infection spot to protect the uninfected tissues against pathogen invasion may circulate as an heterocomplex, where methyl-SA binds to lipid derivatives and lipid-transport proteins and moves through the phloem to the rest of the plant. Thus, studies on TMV-infected tobacco plants revealed that MeSA participates to the perpetuation of SAR defense (Park et al., 2007; Dempsey and Klessig, 2012). Recent data suggest that the composition of the mobile immune signal in SAR relies on a complex network of cross-interacting signals (e.g., MeSA, glycerol-3-phosphate, the lipid-transfer protein DIR1 and the amino acid-derivative pipecolic acid), that differs depending on the plant species and the type of plant–pathogen interaction (Vlot et al., 2008; Dempsey and Klessig, 2012; Spoel and Dong, 2012). Jasmonic acid (JA) is also strongly involved in plant defense against viruses and the HR response initiated by Avr-R protein interactions results on a modulation of SA and JA. Although SA clearly acts as a positive regulator of plant resistance to viruses, the role of JA is controversial and remains to be fully elucidated. For example, JA seems to regulate negatively the local resistance to TMV in tobacco (Oka et al., 2013) but is essential for systemic resistance to TMV in *N. benthamiana* (Zhu et al., 2014). It is likely that a balance between endogenous JA and SA play a key role for determining the degree of resistance, in a similar way to pathosystems involving non-viral plant pathogens (Thaler et al., 2012). Interestingly, plant viruses have evolved targeting hormone pathways, often exploiting the antagonistic interactions between SA and JA pathways (Kazan and Lyons, 2014). Although their function in plant–virus interactions remains poorly understood, recent studies indicate that other plant hormones modulate antiviral resistance mechanisms, especially abscisic acid (Chen et al., 2013; Alazem et al., 2014; Seo et al., 2014), ethylene (Fischer and Dröge-Laser, 2004; Love et al., 2007; Zhang et al., 2009; Chen et al., 2013), and brassinosteroids (Ali et al., 2014).

## CROP IMPROVEMENT AND VIRAL PEST MANAGEMENT: MODERN TECHNOLOGY TO THE RESCUE

Ultimately, the main objective of research on plant–virus interactions consists on the implementation of efficient antiviral resistances in crop plants. For long, genetics-mediated resistance strategies have involved exploiting plant natural variability by introgression of resistance genes through a classical breeding process. However, the onset of evolved pathogens able to overcome these resistance, sometimes very rapidly after resistance deployment (García-Arenal and McDonald, 2003), raised the problem of crop resistance durability and thus the urge to develop new breeding strategies. The last decades saw the emergence of new approaches combining modern technology and state-of-the-art knowledge on plant–virus interactions toward crop improvement programs, triggering a new green revolution in agriculture.

### TECHNICAL ASSETS FROM MARKER-ASSISTED SELECTION

The conventional breeding represents a laborious and time consuming process. In this context, the advent of DNA marker techniques such as random amplified polymorphic DNA (RAPD), restriction fragment length polymorphism (RFLP), amplified fragment length polymorphism (AFLP), and simple sequence repeats (SSRs) contributed, through what is called “Marker-assisted selection (MAS),” to make substantially easier, quicker, and more accurate the selection of resistant genotypes during the introgression steps (Collard and Mackill, 2008). Importantly, this progress has recently been driven by next generation sequencing- (NGS-) based technologies successfully employed for *de novo* whole genome shotgun (WGS) sequencing of reference genotypes and whole genome resequencing (WGRS) of several cultivars, land races, and wild relatives (Mascher and Stein, 2014). Despite the fact that the creation of improved varieties via the MAS strategy is still at its infancy (Collard and Mackill, 2008), MAS has an enormous potential and represents a great challenge of molecular breeding in the 21st century.

### EXPLOITING RESISTANCE-ASSOCIATED QTLs

It is probable that only a small proportion of the natural biodiversity available for disease resistance has been exploited so far. Although more than 80% of reported plant resistances to viruses are monogenically controlled, most agronomic traits in crop plants do not segregate as single defined qualitative monogenic characters but as quantitative and polygenically controlled traits (Maule et al., 2007). Mapping QTL for quantitative resistance requires large sized progenies, nearly saturated genetic maps, as well as reliable and quantitative phenotyping procedures. To date, relatively few QTLs analyses have been performed in plant–virus interactions compared to other pathogens (Maule et al., 2007; Palloix and Ordon, 2011). It is a general assumption that the greater the number of mutations required for virus virulence, the more durable is the resistance. Consequently, the higher durability of polygenic resistances in plant–virus interactions is commonly hypothesized and has recently been validated in the case of pepper resistance to *Potato potyvirus Y* (Palloix et al., 2009; Quenouille et al., 2013). Notwithstanding their clear relevance, QTLs present particular technical challenges for their characterization as well as their incorporation into crops, leading them to be mostly dropped in favor of approaches using monogenic resistances or combining several known major genes.

### GENE PYRAMIDING STRATEGY

The concept of transferring several characterized resistance genes into one plant is called “Gene Pyramiding.” The dogma behind this strategy is that the probability of a pathogen mutating to “virulence against all resistance genes in the pyramid would be the product of the probabilities for each gene singly” (Mundt, 2014), thus making the probability of a virulent pathotype arising highly unlikely. Hence, pyramiding relies on resistance genes that have been previously characterized singly and whose functions are combined within the same plant. This strategy has been successfully applied to plant–virus interactions (Werner et al., 2005; Palloix et al., 2009; Shi et al., 2009) and could bring the opportunity to associate different types of antiviral plant resistances targeting different virus-derived molecules (proteins or nucleic acids) and various processes during virus cycle (replication/translation, plasmodesmata crossing, systemic colonization), with the possible consequence to reduce significantly the probability of resistance breaking by new virus variants (Quenouille et al., 2013). Despite arduous population sizes required for multiple resistance genes along with other agronomical traits, gene pyramiding is gaining considerable importance in plant–pathogen interactions and thus is representing an ambitious challenge for crop improvement programs.

### ENGINEERING RESISTANCES

Genetic engineering directly manipulates the genome of a plant by inducing the expression of novel proteins or by modulating the expression of targeted genes. The genetically modified (GM) plants resistant to viral attacks are grouped into three major categories. First of all, resistance genes with a dominant determinism can be introduced to another plant species. The best examples in plant–virus interactions involve the successful transfer of *R* genes into solanaceous related plants (Whitham et al., 1996; Bendahmane et al., 1999; Baurès et al., 2008; Candresse et al., 2010) but it seems also possible across plant families (Seo et al., 2006). The opportunity to introduce *R* genes from sexually incompatible species is, however, weakened by the fact that most *R* genes display a limited-spectrum resistance. Alternative transgenic approaches have also been developed based on the integration of a viral protein or genomic region into the host plant, through a phenomenon called pathogen-derived resistance (PDR). Initially reported in transgenic tobacco plants expressing the TMV CP (Abel et al., 1986), the resistance observed was ultimately ascribed to the action of the transgene-encoded CP, which disturbs the disassembly of incoming TMV particles. In many other studies, resistance was obtained through the expression of partial or non-coding viral sequences. This kind of resistance, more efficient that the protein-mediated resistance, was later shown to be based on the RNAi mechanism (Tenllado et al., 2004). These observations led to a plethora of RNAi-mediated engineered resistances, using virus-derived double-stranded, hairpin RNAs, or artificial miRNAs (e.g., Qu et al., 2007; Hashmi et al., 2011; Shimizu et al., 2011; Yadav et al., 2011; Zhang et al., 2011a,b; Lin et al., 2012; Shekhawat et al., 2012; Lemgo et al., 2013; Odipio et al., 2013; Zhao and Song, 2014). In the cases where virus-resistant GM varieties have been deployed and/or commercialized (including transgenic squash, papaya, plum, potato, and bean), this strategy has so far proven to be remarkably efficient (Tricoli et al., 1995; Lius et al., 1997; Ferreira et al., 2002; Malinowski et al., 2006; Zagrai et al., 2008; Bravo-Almonacid et al., 2012; Aragão et al., 2013). In spite of engineering resistance success (especially RNAi-based resistant crops), public concerns over the potential ecological impact of GM crops and GM organisms overall have so far significantly limited their use, in particular in Europe.

### THE TILLING AND ecoTILLING REVOLUTION

The early 2000s have seen the emergence of the TILLING (Targeting Induced Local Lesions IN Genomes) method, that consists of a classical mutagenesis step followed by the targeted search for plants carrying a mutation in a gene of interest (McCallum et al., 2000). By creating artificial polymorphism directly into crops, this technique allows potentially: (i) to replace a resistance allele overcome by viral strains, (ii) to generate novel resistances with a broader spectrum, or (iii) to create a new resistance gene based on knowledge acquired in heterologous systems. Such a strategy increase the natural allelic diversity by the identification of novel artificial alleles. Although this strategy requires the previous characterization of the gene conferring the resistance, the main advantage of TILLING is that it can be applied to any plant species, regardless of its genome size, ploidy level or method of propagation, and without introducing heterologous DNA as for GM plants. Similarly, the TILLING natural alternative (using natural germplasms collections instead of EMS mutants collections) is calling ecoTILLING and consists of exploiting the whole natural variability of a plant species (including wild-related and cultivated genotypes; Comai et al., 2004). Initially developed in *A. thaliana*, both TILLING and ecoTILLING have spread rapidly to other model plants (*Medicago truncatula*, *Lotus japonicus*, *Brachypodium distachyon*) and major crops (e.g., maize, soybean, sorghum, tomato, pepper, cucumber, pea, wheat, banana, bean, rice, barley, *Brassica napus*). It seems now clear that these strategies are emerging as major crop improvement tools, with especially successful examples of recent applications to antiviral protection (Nieto et al., 2007; Piron et al., 2010).

### CHEMICAL TREATMENTS

Curative treatments for virus control in fields is impossible. However, chemical applications can prime plant defense, i.e., resistance mechanisms are switched on prior to future infections. Based on the findings that exogenous applications of plant hormones trigger an efficient and broad-spectrum resistance to viral and non-viral pathogens, many SAR-priming molecules have been characterized. Among a series of SA analogs (Gozzo and Faoro, 2013), benzothiadiazole (BTH) has been identified as the safest and most efficient SAR activator and was brought to the market with the common name of acibenzolar-*S*-methyl, that leads to plant protection against many pathogens including viruses (Friedrich et al., 1996; Lawton et al., 1996; Mandal et al., 2008; Takeshita et al., 2013; Trejo-Saavedra et al., 2013). This priming approach represents an environment-friendly and efficient way to control plant diseases by exploiting a natural phenomenon. Despite these positive aspects, so far it has not yet been met with enthusiastic favor by the farmers, as the treatment efficacy with BTH (as well as other SAR-priming molecules) depends on many variables: dose, plant species and cultivar, growth stage of plant, pathogen pressure, climatic conditions and timing of chemical applications (Gozzo and Faoro, 2013). Nevertheless, coupled to genetic strategies, chemical SAR priming is still a valuable method to increase plant resistance against viruses for pest management programs in fields.

### PLANT VACCINATION

Plant vaccination gambles on cross-protection, a phenomenon whereby the inoculation of a virus into a host plant prevents the multiplication of a subsequent challenge virus. This strategy mainly relies on the manipulation of the primary virus, whose infection is weak (symptomless with low viral load), and that triggers virus-induced gene silencing (VIGS) targeting both primary and challenge viruses (Ziebell and Carr, 2010; Nishiguchi and Kobayashi, 2011). Primary viruses act as vaccines and are classified into two categories: the attenuated and the engineering viruses. An attenuated virus corresponds to a weak isolate that triggers cross-protection against virulent isolates of the same virus or closed related viruses (Ziebell and Carr, 2010; Nishiguchi and Kobayashi, 2011). Many examples of cross-protection have been identified since the discovery of this phenomenon by McKinney in 1920s (McKinney, 1926; Crowdy and Posnette, 1947; Fletcher, 1978; Wang, 1991; Wen et al., 1991; Hugues and Ollennu, 1994; Valkonen et al., 2002; Kurihara and Watanabe, 2003; Ichiki et al., 2005; You et al., 2005; Kosaka et al., 2006; Yoon et al., 2006; Ziebell et al., 2007; Nakazono-Nagaoka et al., 2009; Kurth et al., 2012). A great illustration of this method involves an attenuated isolate of *Zucchini yellow mosaic potyvirus,* that has since been registered as the pesticide CUBIO ZY-02 and successfully employed in virus disease control in Japan (Kosaka and Fukunishi, 1997; Kosaka et al., 2009). Moreover, tristeza disease caused by CTV is currently controlled by mild CTV isolates, which when inoculated into existing field trees, extend the productive life of orchards and enable a more graduate replanting of trees on CTV-tolerant rootstocks (Lee and Keremane, 2013). However, identifying effective attenuated viruses for each virus of economical importance might be very arduous. A possible solution came recently from the development of engineering vaccines based on viral vectors carrying a genomic fragment of the virulent virus of interest (Culver, 1996; Bazzini et al., 2006; Tamura et al., 2013; Taki et al., 2013). Given *Apple latent spherical cheravirus* (ALSV) is able to infect a broad range of herbaceous crops (e.g., tomato, lettuce, zucchini, spinach, soybean, tobacco) and fruit trees (e.g., apple, cherry, peach, plum, pear, and citrus) without causing any symptoms, this virus was identified as an excellent candidate for VIGS-mediated vaccination, that could be simply applied widely by replacing the insert with a sequence derived from a different virus. This strategy has been recently illustrated (Tamura et al., 2013; Taki et al., 2013) and represents a very promising technology in virus epidemics management in fields.

## NEW RESEARCH LEAD: LET’S TALK ABOUT ANTIVIRAL PTI IN PLANTS

In an attempt to increase resistance durability in fields, crop improvement requires a continuous pipeline of new resistance genes. Given the importance of PAMP-triggered immunity (PTI) in the field of pathology over the last decades, PTI against plant viruses represents an unexplored question that needs to be addressed.

### PLANT INNATE IMMUNITY

Over the last decades, a concept revolutionizing the understanding of immunity emerged in plant pathology. This new concept stems from the ability for each organism to discriminate between self and non-self molecules through the action of pattern recognition receptors (PRRs) perceiving specific microbial molecular signatures, named pathogen-associated molecular patterns (PAMPs). Perception of PAMPs by these immune receptors induces a downstream signaling cascade including PRR association with positive regulators, phosphorylation events, successive activation of cytoplasmic kinases (including the MAP kinases) and defense-related transcription factors, as well as specific defense genes expression. This range of fast, efficient and multi-layered defense responses is referred as PTI (Nicaise et al., 2009; Macho and Zipfel, 2014). To counteract this defense strategy, successful pathogens deploy effectors proteins, the primary function of which is to evade/interfere with PTI. In turn, some plant cultivars have evolved R proteins to block these effectors, leading to effector-triggered immunity (ETI; Jones and Dangl, 2006). Because both PTI and ETI defense layers rely on cellular actors already present primary to the infection, they are commonly classified into plant innate immunity, in opposition to the concept of adaptive immunity (e.g., gene silencing), where the defense responses are acquired following an infection and are adapted to the pathogen.

### IT’S ALL ABOUT GOOD QUESTIONS

To date, most current models about plant innate immunity exclude viruses, as they are not generally viewed as encoding typical PAMPs and effectors that would trigger PTI and ETI responses (Jones and Dangl, 2006; Boller and Felix, 2009; Schwessinger and Ronald, 2012; Dangl et al., 2013). The fact that virus biology differs greatly from other pathogens raises several questions of importance: could plants defend themselves against viral attacks through similar defense mechanisms employed against non-viral pathogens? And then, could the zig-zag model from Jones and Dangl (2006) be applied to plant–virus interactions?

Given that *R* gene-based immunity in plant–virus interactions involves specific recognition of viral avirulence factors by host-encoded NBS-LRR proteins and leads to HR and SAR (described previously in this review), recent hypotheses advance that it may correspond to antiviral ETI mechanisms (Moffett, 2009; Mandadi and Scholthof, 2013; De Ronde et al., 2014; Nakahara and Masuta, 2014). If so, it may suggest that viral avirulent factors perceived by R proteins correspond to pathogenic effectors, the primary function being to target PTI. Therefore, the concept of antiviral innate immunity as a whole lies mainly in whether it exists virus-associated PRR pathways and viral-encoded effectors interfering with PTI responses. Therefore, the question to assess in priority is whether plant viruses possess molecular features meeting the definition of PAMPs.

### DO VIRUSES ENCODE PAMPs?

Pathogen-associated molecular patterns are defined as highly conserved molecular signatures, characteristic of entire classes of microbes and with an essential function for these microbes (Medzhitov and Janeway, 1997), which implies that they are under strong evolutive pressure and thus, not easily modified by mutations. Many PAMPs from plant pathogens have been identified and so far, they are comprised of proteins, lipids, or carbohydrates (Boller and Felix, 2009). Virus genomes are highly variable even within the same viral family and display high mutation rates during the infection process (≥1 mutation per genome per round of replication), making the expression “viral populations” more suitable than “viruses” when speaking of infected hosts. This elevated mutation rate allows viruses to quickly adapt to changes in their host or environment. Therefore, the PAMP definition referring to small molecular motifs conserved within a class of pathogen is hardly applicable to viral protein motifs. By contrast, virus genomes and their replicative forms (such as dsRNAs) display structural characteristics distinguishing them from the nucleic acid sequences from prokaryotes and eukaryotes. Consequently, these very conserved nucleic acids-composed features could be perceived as non-self signals in plants, acting thus as viral PAMPs.

### WHAT DO WE KNOW ABOUT PTI IN ANIMAL–VIRUS INTERACTIONS?

In the animal field, the most prominent group of PRRs comprises the Toll-like receptors (TLRs), a family of a dozen type I-transmembrane proteins containing a domain with LRRs and a tail that contains a conserved region called Toll/IL-1 receptor (TIR) domain (Beutler, 2004), and sharing common recognition strategies and structural features with plant PRRs (Zipfel and Felix, 2005; Ronald and Beutler, 2010). The TLRs involved in antiviral PTI are localized in endosomes and perceive pathogen nucleic acids: TLR3 binds virus-derived dsRNA, TLR7 and TLR8 recognize viral single-stranded RNAs (ssRNA) and TLR9 mediates the perception of unmethylated CpG DNA from viruses (as well as bacteria and protozoa; Beutler, 2004). After PAMP perception, the activated receptor seems to form homo- and/or hetero-dimers with other TLRs. The TIR domains of these complexes recruit cytoplasmic adaptors, triggering a signaling cascade that mainly consist on the successive activation of cytoplasmic kinases including the MAP kinases, defense-related transcription factors and the expression of specific defense genes (Beutler, 2004; Kawai and Akira, 2008; Sandig and Bulfone-Paus, 2012). In addition, subsequent studies revealed that TLR-independent recognition of viruses can be also accomplished by cytosolic PRRs, including the two RNA helicases RIG-I (*Retinoic acid-inducible gene I*) and MDA5 (*Melanoma differentiation-associated gene 5*), both highly activated by dsRNAs, as well as 5′ triphosphate-RNAs for RIG-I (Kawai and Akira, 2008). In the arm race between host and microbes, successful animal viruses have evolved to suppress and/or hijack PTI responses, leading to host colonization. This overcoming of defense mechanisms is orchestrated by virulence effectors encoded by virus genomes. Among the strategies employed by viruses to suppress host immunity, inactivation of cytoplasmic kinases, inhibition of PRR complexes, stabilization of immunity-related negative regulators and inactivation of transcription factors are particularly noteworthy (Schröder and Bowie, 2007; Yokota et al., 2010).

### ON THE TRAIL OF PTI AGAINST PLANT VIRUSES

The existence of animal PRRs specific to viral PAMPs raises the question of the existence of PRRs perceiving plant viruses. Although no PTI pathways against plant viruses has been formally characterized to date, typical PTI cellular responses are observed during plant–virus interactions, including ion fluxes (Shabala et al., 2010, 2011; Otulak and Garbaczewska, 2011), ROS production (Allan et al., 2001; Love et al., 2005; Díaz-Vivancos et al., 2008), ethylene signaling (de Laat and van Loon, 1982; Love et al., 2005), and callose deposition (Iglesias and Meins, 2000; Li et al., 2012; Zavaliev et al., 2013). Key components of plant PTI pathways are also involved in antiviral defense mechanisms. SA, known to regulate innate immunity including PTI responses (Tsuda et al., 2008; Yi et al., 2014), plays an important role in plant–virus interactions (Carr et al., 2010). MAPK4, that regulates negatively plant PTI mechanisms (Gao et al., 2008; Kong et al., 2012), represses soybean defense against *Bean pod mottle comovirus* (Liu et al., 2011). The receptor-like kinases (RLKs) BAK1 (BRI1-Associated receptor Kinase 1) and BKK1 (BAK1-like Kinase 1), key activators of plant PRRs after PAMP perception (Kim et al., 2013), are required for *Arabidopsis* resistance to plant viruses (Yang et al., 2010; Kørner et al., 2013). Moreover, the protein NIK1, another RLK highly similar to BAK1 and BKK1, has been strongly associated with *Arabidopsis* defense against the *Cabbage leaf curl geminivirus* (CaLCuV). Strikingly, NIK1 is cleaved by the viral protein NSP during the infection, suppressing its kinase activity and thus enhancing susceptibility to CaLCuV infection (Fontes et al., 2004; Carvalho et al., 2008). Overall, these data support the hypothesis that plants may defend themselves against viruses through similar PTI pathways employed against non-viral pathogens.

Accumulating data indicate that: (i) colonization strategies employed by plant and animal viruses inside host cells are highly similar (Thivierge et al., 2005; Taliansky et al., 2010; Nicholson and White, 2014), (ii) plant and animal PTI mechanisms display impressive structural and functional similarities (Zipfel and Felix, 2005; Dardick and Ronald, 2006; Ronald and Beutler, 2010), (iii) effectors from animal and plant pathogens target similar PTI components (Schröder and Bowie, 2007; Yokota et al., 2010; Deslandes and Rivas, 2012) and, (iv) plant defense against viruses involves many components belonging to PTI signaling (described above). Therefore, the apparent universality of PTI strategies identified so far in eukaryotic organisms enable an extrapolation of antiviral pathways from animal toward plant models.

**FIGURE 3:**
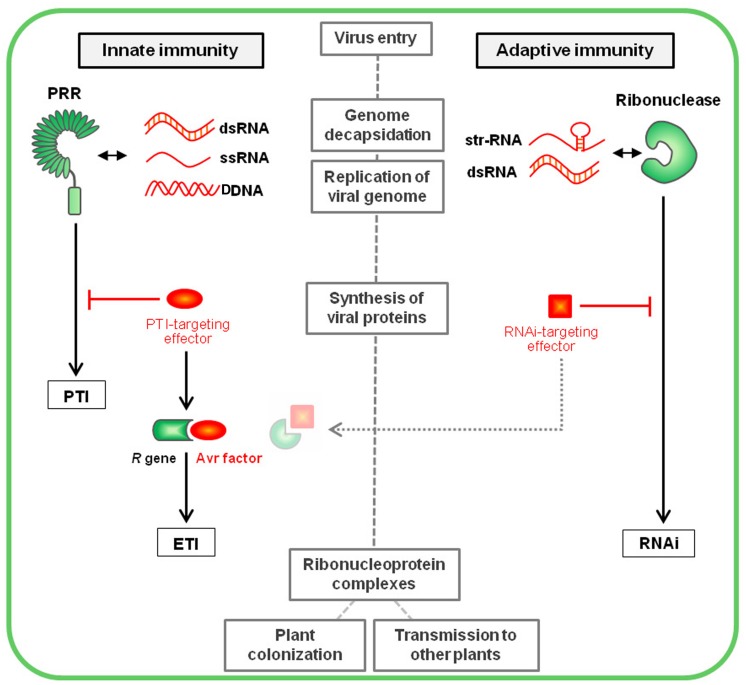
**Plant innate immunity vs. Plant adaptive immunity.** Following entry into a host cell and genome decapsidation, the virus genome is liberated and replicated, leading to the accumulation of pathogenic nucleic acids that are perceived as viral PAMPs by specific intracellular PRRs. This recognition triggers a downstream cascade leading to PTI responses. Virus genome translation (occurring simultaneous to replication) leads to the synthesis of the virus-encoded proteins, among which viral pathogenic effectors enable the suppression of PTI signaling. In turn, specific plant *R* genes interact (directly or indirectly) with these effectors (that are then called avirulence factors) to trigger ETI. In the case of plant adaptive immunity illustrated by RNA interference (RNAi), the virus-derived elicitor molecules, corresponding to replicative dsRNAs or structured (str-RNA) viral genomes, are recognized by DCLs, key component of the silencing machinery that leads to virus degradation at the nucleic acid level. Viral proteins acting as RNAi-suppressing effectors interfere with this defense pathway. A recent publication (Sansregret et al., 2013) suggests silencing suppressors may be targeted by ETI-like mechanisms, restoring plant resistance. The main steps of the virus cycle into the first infected cell have been mentioned into gray boxes.

Thus, strongly based on the understanding of plant immune pathways and the knowledge on antiviral mechanisms from the animal field, I propose here a model of plant immunity against viruses (**Figure 3**), that does not invalidate previous models but instead tries (i) to bring the concept of PTI as a novel plant antiviral mechanism, and (ii) to integrate the concept of innate (PTI and ETI) and adaptive (gene silencing) antiviral immunities, both triggered by similar virus-derived elicitor molecules, in order to address the important question of plant defense against viruses in a more holistic way.

Thus, following the virus entry into a plant cell, viral nucleic acids are perceived as PAMPs by specific intracellular PRRs triggering PTI pathways (**Figure 3**). Successful viruses encode specialized effectors that are able to suppress this defense layer. In turn, specific *R* gene products interact with these effectors (playing then the role of avirulence factors), leading to ETI. In the case of RNAi-based adaptive immunity, the viral elicitor molecules (mostly dsRNAs) are recognized by DCLs. This leads to virus degradation at the nucleic acid level, a process that can be suppressed by a second class of virus-encoded effectors, commonly named silencing suppressors. Interestingly, Sansregret et al. (2013) recently highlighted a resistance mechanism antagonizing the silencing suppressor P19, through a phenomenon of extreme resistance characterized by a SA- and ethylene-dependent process without microscopic cell death. Although the reliance of this mechanism on one or several R proteins remains to be established, this data suggest that the action of RNAi-suppressing effectors might be countered by ETI-like mechanisms.

It is important to note here that recent reviews refer DCLs themselves as PRRs perceiving viral nucleic acids and triggering immune responses equivalent to the zig-zag model first layer (Pumplin and Voinnet, 2013; Sansregret et al., 2013; Nakahara and Masuta, 2014). Of course, PTI and RNAi mechanisms are both induced by highly similar virus-derived molecules that both fit the definition of PAMPs, suggesting that these virus-derived molecules trigger two different pathways in parallel (PTI and RNAi), similarly as in the animal field (Yan and Chen, 2012). In line with this, DCL-mediated immunity should maybe be called PTI too. However, this idea faces a conceptual and semantic conflict, as the classical view of PTI lies on innate immunity, while silencing triggers an immunity that is classified as adaptive. This issue could be partially solved by raising the concepts of innate and adaptive PTIs, that both would be targeted by plant ETI. This notwithstanding, this situation reveals that these processes represents two distinct but tight and intricate branches of the whole plant immune arsenal against viruses, raising important conceptual questions that will need to be answered.

Indeed, future investigations will need first to bring the clear proof of the existence of PTI pathways triggered by the recognition of viral PAMPs by plant PRRs. To this perspective, the identification of pathogenic effectors from successful plant viruses that target specifically PTI responses will be definitely an asset, as pathogenic effectors represent fantastic molecular tools commonly used to characterize innate immunity signaling.

### WHICH ADVANTAGE PTI COULD BRING INTO CROP IMPROVEMENT PROGRAMS?

So far, the only powerful strategy to control viral epidemics in field relies on the use of genetic resistances. Unfortunately, viruses evolve very quickly the ability to overcome the resistances employed by breeders. Hence, handling the extreme genetic plasticity of viruses represents a major challenge for the coming years. The durability of plant resistances, even more with viruses than any other plant pathogen, depends mostly on the resistance timing/efficiency and on the elicitor nature/conservation within pathogen families, in order to enable an efficient, sustainable, and broad-spectrum resistance.

Given their importance for pathogen survival, PAMPs are under a strong evolutive pressure and are highly conserved within pathogen families, this definition fitting perfectly with the characteristics of viral nucleic acids, whose features (e.g., dsRNAs replicative forms, highly structured genomes) are highly conserved within virus families and differ radically from prokaryotic or eukaryotic nucleic acids. It is these typical viral features that are perceived by specialized PRRs in animals (e.g., TLRs), triggering very fast and very efficient immune responses. Because the innate pathway is solely programmed by structural and nucleotide sequence genomic features, plant antiviral PTI should be a remarkably versatile mechanism because it could respond virtually to any plant virus. Moreover, timeliness of defense responses is a key parameter in resistance success, especially in the case of viruses, given their tremendous adaptation abilities. And yet, the first measurable PTI responses are within minutes after PAMP perception (Nicaise et al., 2009), making PTI the quickest plant defense identified so far. Hence, translating fundamental knowledge about PTI mechanisms for new crop resistance deployment must bring considerable advances in the control of viral epidemics in fields. Moreover, what is increasingly clear is that plant antiviral arsenal ensure a highly robust plant immune system, through different plant defense layers (R genes, SAR, RNAi, PTI, and ETI) that emerge to be finely coordinated (Voinnet, 2005; Alamillo et al., 2006; Boccara et al., 2014; Padmanabhan and Dinesh-Kumar, 2014). Consequently, addressing plant–virus interactions holistically by combining PTI-mediated resistance with other defense pathways (such as silencing) could provide highly robust resistance phenotypes targeting virus cycle as a whole.

## CONCLUSION

In the world agriculture context, intensification of cultural practices, climate alterations, and extensive exchanges affecting global markets are associated with an increased incidence of plant viral diseases. In consequence, management of viral epidemics is a matter of global food security. Nowadays, antiviral measures employed in fields benefits greatly from the latest knowledge highlighted by plant virologists, geneticists and molecular biologists, as well as engineers and breeders, whose coordinated effort enables a deployment in crop improvement programs. This notwithstanding, the situation in fields remains worrying, given the emergence of numerous crop diseases ascribed to viruses, a statement that could be aggravated in the future through climate changes that might affect strongly virus and organism-vectors populations in terms of magnitude, locations and dynamics (Anderson et al., 2004; Gautam et al., 2013). In a coordinated manner with technological advances, fundamental research needs to explore new scientific leads, deciphering more and more thoroughly the intricate molecular dialog between a plant virus and its host. Hence, future challenges associated to the management of crop viral diseases will rely mainly on integrated research actions with a view to translating fundamental understanding toward applied programs, and thus reducing the gap existing between the laboratory and the field.

## Conflict of Interest Statement

The author declares that the research was conducted in the absence of any commercial or financial relationships that could be construed as a potential conflict of interest.
